# Menopause, obesity and inflammation: interactive risk factors for Alzheimer’s disease

**DOI:** 10.3389/fnagi.2015.00130

**Published:** 2015-07-07

**Authors:** Amy Christensen, Christian J. Pike

**Affiliations:** Davis School of Gerontology, University of Southern CaliforniaLos Angeles, CA, USA

**Keywords:** adiposity, aging, Alzheimer’s disease, estrogen, hormone therapy, inflammation, obesity

## Abstract

Alzheimer’s disease (AD) is a multifactorial neurodegenerative disorder, the development of which is regulated by several environmental and genetic risk factors. Two factors theorized to contribute to the initiation and/or progression of AD pathogenesis are age-related increases in inflammation and obesity. These factors may be particularly problematic in women. The onset of menopause in mid-life elevates the vulnerability of women to AD, an increased risk that is likely associated with the depletion of estrogens. Menopause is also linked with an abundance of additional changes, including increased central adiposity and inflammation. Here, we review the current literature to explore the interactions between obesity, inflammation, menopause and AD.

## Introduction

Alzheimer’s disease (AD) is an age-related neurodegenerative disease that is the leading cause of dementia. The causal factor(s) that drives development and progression of the disease is still debated, though the primary agents are likely β-amyloid protein (Aβ) and the microtubule associated protein tau (LaFerla, 2010; Morris et al., 2014). Aβ is a small, soluble peptide normally found at low levels in brain and bodily fluids. Increased production and/or decreased clearance of Aβ fosters its inherent ability to self-associate into neurotoxic oligomeric forms that become deposited in the brain parenchyma as plaques and in the cerebrovasculature as cerebral amyloid angiopathy (Mucke and Selkoe, 2012). Interestingly, genetic mutations that underlie familial AD yield increased Aβ production or its propensity to aggregate (Tanzi, 2012). Thus, given both the toxic nature of Aβ and its genetic links to the disease, Aβ accumulation is widely theorized to be the key regulator of AD pathogenesis (Hardy and Higgins, 1992; Hardy, 2006). However, increasing evidence indicates an essential role of tau, which undergoes hyperphosphorylation resulting in the formation of neurofibrillary tangles, a hallmark of AD neuropathology found in many dead and dying neurons (Iqbal et al., 2010). Emerging evidence indicates that tau, like Aβ, can be a potent pathogenic protein and that it is capable of spreading pathology in a prion-like manner (Bloom, 2014; Zempel and Mandelkow, 2014).

AD is more than just the accumulation of oligomeric and fibrillar Aβ and abnormally phosphorylated tau. The disease is characterized by many pathologic changes, including hypometabolism (Mosconi et al., 2008; Yao et al., 2011), blood-brain-barrier (BBB) disruption (Zlokovic, 2011), and glial activation (Mrak and Griffin, 2005; Prokop et al., 2013). Sporadic AD, which is not driven by the genetic mutations in familial AD and represents the vast majority of cases, is likely to reflect the interactive effects of normal aging with numerous environmental risk factors and subtle genetic polymorphisms. In turn, these interactions cooperatively disrupt pathways that regulate Aβ, tau, and other AD pathologies. Thus, unraveling AD risk, and perhaps developing successful prevention and intervention strategies, will require understanding the interactions between aging and a set of risk factors.

Two important AD risk factors are obesity and inflammation. Obesity has increased at an alarming rate across westernized countries, with approximately 70% of the US adult population currently classified as either overweight or obese (Ogden et al., 2014). Elevated adiposity increases the risks of numerous conditions, including metabolic syndrome (McGill, 2014), hypertension (Rahmouni, 2014), cardiovascular disease (Rocha and Libby, 2009), and AD (Jayaraman and Pike, 2014). One consequence of obesity that may underlie its pathogenic roles is chronic inflammation, which is observed both in brain and systemically. Inflammation is independently associated with AD and numerous other age-related disorders (Michaud et al., 2013; Bettcher and Kramer, 2014). A wealth of genetic, epidemiological, and experimental findings identifies pro-inflammatory pathways as significant regulators of various AD pathologies (Wyss-Coray and Rogers, 2012). Interestingly, risk factors can affect men and women differently. For example, the apolipoprotein E ɛ4 allele (ApoE4), the most significant genetic risk factor for late-onset AD, affects women much more strongly than men (Altmann et al., 2014). The enhanced vulnerability of aging women to some conditions, including increased risk of osteoporosis, cardiovascular disease and perhaps AD, is related to depletion of estrogens at menopause. Here, we consider the roles of obesity and inflammation in AD pathogenesis with a specific emphasis on women, who are disproportionately affected by AD.

### AD and Obesity

Obesity is a precursor condition for numerous disorders, including cardiovascular disease, metabolic syndrome, and type 2 diabetes (T2D; Bonomini et al., 2015; Kim and Feldman, 2015). More recently, obesity and its associated comorbid conditions have been identified as significant risk factors for both cognitive decline and the development of AD (Jayaraman and Pike, 2014). A growing literature suggests that the insulin resistance and dysregulation of insulin signaling associated with T2D are precursors to both cognitive impairment and AD (Yaffe et al., 2004; Profenno et al., 2010). T2D in humans has been shown to increase the rate of age-related mental decline (Hassing et al., 2004), and those with T2D have been shown to develop cognitive impairment earlier than those without this risk factor (Cigolle et al., 2011). Further, adults with T2D have a significantly increased risk of AD (Luchsinger et al., 2004; Biessels et al., 2006; Profenno et al., 2010). Similarly, obesity appears to significantly increase AD risk, particularly when it is present during middle age (Kivipelto et al., 2005; Beydoun et al., 2008; Fitzpatrick et al., 2009; Xu et al., 2011). In contrast to most of the literature, a recent study with a very large sample size reported that midlife obesity (measured as body mass index, BMI) reduces dementia risk (Qizilbash et al., 2015). The absence of concordance among several well-controlled studies suggests that the relationship between obesity and AD is more complex than simply an increase in adiposity. For example, some studies suggest that dementia risk is not adversely affected by BMI *per se*, but rather by central obesity specifically (Whitmer et al., 2008; Gustafson et al., 2009; Luchsinger et al., 2012), which is often closely associated with adverse health effects including cardiovascular disease. In fact, cardiovascular outcomes are predicted better by measures of central obesity and often poorly by BMI (Yusuf et al., 2005; Dallongeville et al., 2012). Age is also an important consideration, as obesity in late life appears to reduce rather than increase AD risk (Fitzpatrick et al., 2009; Gustafson et al., 2009). Collectively, these observations suggest that central obesity in midlife induces changes that may create a neural environment conducive to initial development of AD pathology.

In general agreement with the human literature, experimental studies in mouse models of AD demonstrate that diet-induced obesity (DIO) significantly exacerbates AD-like neuropathology and worsens cognitive impairment (Julien et al., 2010; Herculano et al., 2013). Short-term high fat diet in an AD mouse model was shown to cause mild metabolic dysfunction and significant cognitive impairment, although no changes were observed in levels of Aβ, a key protein in AD pathogenesis (Herculano et al., 2013). High fat and high sucrose diets have also been shown to affect tau accumulation, processing and hyperphosphorylation (Julien et al., 2010; Orr et al., 2014; Takalo et al., 2014). In 3 × Tg-AD mice, longer-term administration of a high fat diet (16 weeks) impaired cognitive performance in both male and female mice (Barron et al., 2013). In line with the behavioral deficits, both male and female 3 × Tg-AD mice showed increased Aβ burden in the hippocampus. Male and female non-transgenic rodents both exhibit decreased cognitive performance after exposure to high fat diet (Molteni et al., 2002; Farr et al., 2008; Granholm et al., 2008). In some cases, male mice are more vulnerable to diet-induced cognitive impairment (Hwang et al., 2010). Since males typically exhibit significantly more robust metabolic deficits in response to high-fat diets (Barron et al., 2013), this may suggest that some aspects of obesity-induced cognitive impairment are related to metabolic disturbances, including insulin resistance. However, since even short-term exposure (9 days) to diets high in fat (Murray et al., 2009) results in impaired spatial memory in rats, more immediate effects of diet must also contribute to cognitive deficits. Diets high in sugars can also yield negative outcomes in AD mouse models (Cao et al., 2007; Orr et al., 2014) and wild-type rats (Hsu et al., 2015). Together, these studies show that exposure to obesity-inducing diets results in both rapid and long-term neural changes that impair cognitive performance and accelerate development of AD-like pathology.

The mechanism(s) by which obesity increases AD risk and cognitive deficits is unknown, although numerous possibilities have been proposed (Jayaraman et al., 2014). One widely discussed concept is that AD risk is linked with changes in glucose metabolism and insulin signaling (Blázquez et al., 2014; de la Monte and Tong, 2014). Consistent with this position, a reduction in brain glucose metabolism has been shown to be a preclinical symptom of AD (Mosconi, 2005). This reduction appears to be associated with altered insulin signaling. In obese patients, insulin resistance results in an elevated release of peripheral insulin, but insulin concentrations in the brain are reduced, likely due to a decrease in insulin transport across the BBB (Craft, 2007). Although insulin levels are decreased centrally, the situation is further exacerbated by altered insulin signaling in the brain that is less effective than under normal conditions (Anthony et al., 2006). Interestingly, intranasal administration of insulin has been shown to improve hippocampal dependent memory in patients with early stages of AD (Reger et al., 2008; Claxton et al., 2015). Perhaps in contrast to these observations, DIO in female 3 × Tg-AD mice accelerated Aβ accumulation and behavioral impairment in the absence of apparent changes in peripheral insulin levels and sensitivity (Barron et al., 2013). Collectively, these data suggest that obesity-induced insulin resistance and/or impaired insulin signaling can contribute to AD neuropathology although additional factors also play significant roles.

Other than insulin-related deficits induced by obesity, increased adiposity has other systemic effects that may contribute to induction and progression of AD. For example, increased adiposity elevates neuroinflammation (Thaler and Schwartz, 2010; Thaler et al., 2012; Jayaraman et al., 2014; Aguilar-Valles et al., 2015), which has been implicated as a pathologic mechanism in AD (Johnston et al., 2011; Verri et al., 2012; Wyss-Coray and Rogers, 2012). Systemic and central inflammation will be discussed in greater detail below. However, cerebrovascular inflammation in particular has been widely associated with both obesity (Tucsek et al., 2014) and AD (Yu et al., 2012; Takeda et al., 2013). In fact, vascular inflammation may precede AD, as a transgenic rodent model of obesity and AD showed cerebrovascular inflammation and cognitive deficits prior to the deposition of Aβ (Takeda et al., 2010). In this model, cerebral amyloid angiopathy was much greater in obese animals compared to lean ones. Other vascular risk factors that are associated with obesity, including hyperlipidemia and hypertension, have also been identified as AD risk factors (Kivipelto et al., 2005). Thus, the mechanisms by which obesity increases AD risk are presumably multifactorial and likely modulated by a constellation of interactive risk factors and comorbidities.

### AD and Inflammation

Although inflammation has been implicated in AD pathogenesis for many years, the importance of its role has been increasingly appreciated in the past few years. As has been discussed in other recent reviews (Mandrekar-Colucci and Landreth, 2010; Wyss-Coray and Rogers, 2012), multiple lines of evidence link inflammation as a key contributor to both the initiation and progression of AD and identify it as a compelling therapeutic target. Much of the recent focus on inflammation comes from the linkage to AD of polymorphisms in genes that are expressed by microglia and or play a role in innate immune responses (Tanzi, 2012). Included in this list are CD33 (Bertram et al., 2008; Hollingworth et al., 2011; Naj et al., 2011) and triggering receptor expressed on myeloid cells 2 (TREM2; Leavy, 2015), which are expressed in microglia and contribute to phagocytosis and Aβ clearance. Other immune-related factors linked to AD are CR1 and clusterin (Harold et al., 2009; Lambert et al., 2009), components of the complement system.

Microglia are the key mediators of neuroinflammation, functioning as the brain’s resident macrophages. In their role as brain immune sentinels, microglia constantly survey the neural environment for both normal and pathological disruptions. In their normal state, microglia are clearly beneficial, utilizing phagocytosis to remove unwanted or unneeded materials and to regulate homeostatic processes such as synaptic remodeling (Chen and Trapp, 2015). Further, they may have important neurotrophic roles as suggested by their secretion of brain-derived neurotrophic factor and insulin-like growth factor 1 (Parkhurst et al., 2013; Suh et al., 2013). When activated under more pathological conditions, microglia exhibit a wide range of responses that continue to be defined (Colton, 2009). Among these responses, microglia alter their phagocytic activities and can secrete large amounts of pro-inflammatory cytokines. Cytokine secretion can be aggravated when microglia are primed by an initial activating insult (Teeling and Perry, 2009). This type of priming response may contribute to interactions among comorbid conditions such as obesity. In this case, a high fat diet that results in obesity may represent the first hit on the microglia and Aβ accumulation the second, yielding an exaggerated response that may drive pathology.

The AD brain is characterized by extensive gliosis, including activation and increased numbers of both astrocytes and microglia as well as elevated levels of inflammatory cytokines. Aβ plaques, once formed, are rapidly (within 1–2 days) surrounded by microglia (Meyer-Luehmann et al., 2008). Aβ has been shown to promote the release of inflammatory cytokines from microglia (Wyss-Coray and Rogers, 2012). Interestingly, increased pro-inflammatory factors can result in greater amyloid precursor protein production, leading to even more Aβ release and creating a vicious cycle that further promotes AD pathology (Karran et al., 2011). This progression of pathology appears to yield multiple states of microglial activation and deactivation that presumably vary in their effects on discrete AD pathologies (Colton, 2009). For example, initial exposures of microglia to Aβ, while the microglia are in their resting anti-inflammatory state, can result in effective Aβ clearance. However, this insult induces an activation state associated with the release of pro-inflammatory cytokines like tumor necrosis factor α (TNFα) and interleukin 1β (IL-1β). After microglia achieve a pro-inflammatory state, they may be less able to effectively phagocytose extracellular Aβ (Koenigsknecht-Talboo and Landreth, 2005). Similarly, microglia in an AD mouse model mice were shown to be anti-inflammatory early in the disease, but to be activated later after the pathology had progressed (Jimenez et al., 2008).

### Obesity and Inflammation

It has been more than two decades since the link between inflammation and insulin resistance was first shown with an increase in TNFα levels in obese rodents (Hotamisligil et al., 1993). Confirming an active rather than passive role in pathology, neutralization of TNFα in obese rats resulted in a restored response to insulin. This and other early studies spawned intense study on the role of numerous inflammatory factors in obesity, metabolic syndrome, and T2D. It is now clear that not only is chronic low grade inflammation a symptom of metabolic disorders, but also that both peripheral (Hotamisligil, 2006) and central (Thaler and Schwartz, 2010) inflammation contribute to the development and progression of obesity and its comorbidities.

Visceral fat, also known as central or abdominal fat, is thought to have the greatest correlation with metabolic dysfunction (Kissebah et al., 1982; Nieves et al., 2003). An increase in body weight results in a corresponding increase in the number of mature macrophages found in adipose tissue. Macrophages invade and surround necrotic adipocytes and secrete excess pro- and anti-inflammatory cytokines, including TNFα and interleukin 6 (IL-6). The release of proinflammatory cytokines by macrophages is thought to contribute to the glucose disruptions and insulin resistance that is seen in obese individuals. Indeed, if macrophages are specifically ablated, insulin and glucose homeostasis can be rapidly restored and levels of inflammatory cytokines in adipose tissue, muscle and serum reduced (Patsouris et al., 2008). Further, adipocytes themselves secrete leptin and IL-6 after the macrophage infiltration and further increase the inflammatory response due in obesity. Adipose tissue is a highly active immunological organ that contributes to the increased inflammatory response to weight gain.

The role of inflammation in obesity also includes key contributions from the brain. Obesity-induced increases in peripheral and circulating cytokine levels likely affect the brain in multiple ways. However, available evidence suggests that central inflammation begins prior to the onset of obesity and appears to contribute to the process (Thaler et al., 2013). Much of the data on central inflammation and obesity has been generated from DIO paradigms, in which increased adiposity results from maintaining rodents on high-fat diets. DIO recapitulates much of the human obesity condition and can be modified to include specialized fats and/or sugars. Rodent DIO models have shown that both central and peripheral inflammation are induced with high fat diet (De Souza et al., 2005; Milanski et al., 2009; Thaler et al., 2012). Inflammation in the hypothalamus in response to high fat diet occurs within hours and persists for a few days before subsiding only to return after about 2 weeks (Thaler et al., 2012). Peripheral inflammation takes longer to develop and usually coincides with an increase in adipose tissue mass. Interestingly, mice with IL-1β, IL-6, or TNFα receptor knocked out show a worsening rather than a reduction in both obesity and metabolic syndrome, suggesting that inflammation is not the only contributing factor to these afflictions.

Inflammatory cytokines, which are elevated by obesity in both adipose tissue and brain, appear to be involved in both promoting inflammation and mediating many of its deleterious effects. In the hypothalamus, DIO induces inflammation within 3 days of the initiation of high fat diet in rodent (Thaler et al., 2012). Short-term high fat diet results in an upregulation of activated microglia and astrocytes. In humans, increased gliosis that is similar to that characteristic of injury is seen in the medial basal hypothalamus of obese patients (Thaler et al., 2012). Consistent with their role in adverse neural outcomes, cortical glia cultured from obese mice retain their inflammatory phenotype and impair neuron survival and growth *in vitro* (Jayaraman et al., 2014). Numerous inflammatory factors have been shown to be upregulated by obesity, including IL-1β, IL-6, TNFα and interleukin 18 (IL-18; Vandanmagsar et al., 2011), but which ones are central the relationship between obesity and AD are unclear. Here, we consider a few of the factors that are affected by obesity with an emphasis on those that also have been implicated in AD.

#### Leptin

Leptin is an adipokine that modulates appetite and informs the brain about the availability of stored energy. Although leptin is thought of as a peptide hormone, it bears remarkable similarity to inflammatory cytokines, especially interleukin 2 (IL-2). Further, the leptin receptor is homologous to the type 1 cytokine receptors. Leptin secretion increases with increasing adiposity (Considine et al., 1996). In the hypothalamus, leptin regulates appetite. High levels of leptin normally induce satiety. However, obese individuals develop leptin resistance, attenuating its ability to properly regulate appetite. The increase in adipose tissue in obese individuals initially results in elevated leptin release, but the response to leptin is blunted and the satiety signal is not recognized. The mechanisms that underlie leptin resistance have not been fully elucidated, but it may be the result of disrupted leptin signaling pathways, decreased leptin transport across the BBB, or increased inflammation in the hypothalamus (Park and Ahima, 2015).

In addition to regulating appetite, leptin is a regulator of inflammation and the immune system. Leptin regulates both innate and adaptive immune responses and generally increases levels of pro-inflammatory cytokines (Conde et al., 2014). Initial evidence of this link emerged from the observation that mice with deficiencies in leptin signaling (*ob/ob* or *db/db*) exhibit impaired immune responses (Lord et al., 1998). On the other hand, leptin exposure increases the proliferation and activation of T lymphocytes and promotes a T helper 1 phenotype (Martín-Romero et al., 2000). Similarly, leptin activates components of the innate immune response. For example, leptin increases activation of monocytes, including their production of cytokines such as TNFα and IL-6 (Santos-Alvarez et al., 1999). Also, leptin can induce the expression of nitric oxide and prostaglandin estrogen 17β-estradiol (E2) in cultured macrophages (Raso et al., 2002). With signaling capacity both peripherally and centrally and release modulated by fat mass, leptin is well positioned to function as a mediator of interactions between the brain and peripheral inflammatory responses (Carlton et al., 2012), particularly in the context of obesity (Aguilar-Valles et al., 2015).

Leptin resistance is predicted to foster AD pathogenesis as normal leptin signaling is associated with reduced AD risk. Persons with mild cognitive impairment or AD show lower plasma levels of leptin than cognitively normal controls (Johnston et al., 2014). Cerebrospinal fluid (CSF) leptin levels can remain stable with progression to AD, but a reduction in leptin signaling in hippocampus suggests AD is associated with attenuation of leptin actions (Maioli et al., 2015). Conversely, in a prospective study, elevated leptin levels were associated with greater cerebral brain volume and reduced dementia risk (Lieb et al., 2009). In experimental AD models, leptin administration has been shown to have therapeutic effects on both Aβ deposition (Fewlass et al., 2004) and tau phosphorylation (Greco et al., 2008). Such protective pathways may become dysfunctional in the AD brain, where leptin levels are elevated but expression of its receptor is downregulated (Bonda et al., 2014). Further, the leptin receptor protein was found localized to neurons containing neurofibrillary tangles and within plaques, a situation expected to disrupt its signaling capability. Indeed, phosphorylation of the leptin receptor, which is required for its activation, was reduced when the leptin receptor was found in tangle-bearing neurons (Bonda et al., 2014). Collectively, available data indicate that a loss of beneficial leptin signaling can contribute to AD, suggesting that leptin resistance may be a mechanism by which obesity affects AD risk.

#### TNFα

TNFα appears to be produced exclusively by macrophages in adipose tissue with no contribution from adipocytes (Weisberg et al., 2003). Both TNFα protein and mRNA are robustly upregulated in obesity, an increase that has been shown to correlate with the development of insulin resistance (Hotamisligil et al., 1995). An increase in abdominal adipose tissue in particular has been shown to correlate with increased TNFα release (Tsigos et al., 1999). After weight loss, a decrease in TNFα protein in adipose tissue and a decrease in serum insulin is observed (Hotamisligil et al., 1995).

The neural effects of TNFα, which is produced in brain by neurons and glia, can be both desirable and detrimental. Transiently elevated levels of TNFα are beneficial. For example, TNFα can recruit astrocytes and microglia to sites of injury and activate a glial response (Flynn et al., 2003). However, TNFα also exerts negative effects on neural plasticity, such as reducing levels of long-term potentiation (Beattie et al., 2002; Ferguson et al., 2008). Increased TNFα is also thought to promote AD pathogenesis. For instance, *in vitro* and *in vivo* evidence indicate that TNFα increases Aβ levels by increasing expression of BACE1, an enzyme that drives Aβ production (Yamamoto et al., 2007). Further, TNFα has been shown to inhibit the transport of Aβ out of the brain and into peripheral circulation where it can be eliminated (López et al., 2008). TNFα-mediated increases in Aβ can lead to a vicious cycle. Aβ not only increases expression of TNFα (Meda et al., 1995; Akama and Van Eldik, 2000) but also is able to activate the TNFα receptor TNFRI, which is upregulated in AD brains compared to normal controls (Li et al., 2004; Cheng et al., 2010). Further, Aβ-induced generation of TNFα is implicated in the neurotoxic effects of Aβ (Xie et al., 2002). Conversely, reducing TNFα may be neuroprotective. For example, TNFRI knockout in AD transgenic mice diminishes AD-like neuropathology (He et al., 2007).

#### IL-6

Both adipocytes and macrophages can generate and secrete IL-6 from adipose tissue. Greater adiposity results in increased IL-6 secretion. Serum IL-6 concentrations have been positively correlated with obesity, insulin resistance (Bastard et al., 2000; Kern et al., 2001), T2D (Pradhan et al., 2001), and cardiovascular disease (Plutzky, 2001). Multiple studies in both mice and humans have linked IL-6 to T2D as the cytokine with the strongest association with metabolic dysfunction (Kern et al., 2001; Pradhan et al., 2001). Although IL-6 production is associated with visceral fat (Fontana et al., 2007), the relationship between IL-6 and obesity is not straightforward. For example, IL-6 knockout mice develop obesity, elevated leptin, and altered glucose homeostasis with aging (Wallenius et al., 2002), suggesting that long-term IL-6 depletion can contribute to metabolic dysfunction. IL-6 administered peripherally in knockout mice, reduces body weight and leptin levels. However, IL-6 treatment is also associated with deleterious effects in several paradigms. For example, in mouse hepatocytes and a human hepatocarcinoma model, IL-6 disrupts insulin signaling by decreasing insulin’s ability to activate Akt, a critical part of insulin’s modulation of downstream metabolic effects (Senn et al., 2002).

As with several other pro-inflammatory cytokines, chronic elevation of IL-6 results in negative neural consequences. Cross-sectional and longitudinal data indicate that elevated plasma IL-6 in mid-life is associated with significant cognitive decline (Singh-Manoux et al., 2014). Several IL-6 genetic variants have been shown to significantly regulate AD risk (Papassotiropoulos et al., 1999; Chen et al., 2012; Flex et al., 2014). IL-6 has consistently been shown to be elevated in the brains, especially near Aβ plaques, and in the CSF of AD patients (Bauer et al., 1991; Blum-Degen et al., 1995). The astrogliosis and microgliosis triggered by AD results in increased IL-6 release from both of these cell types (Erta et al., 2012). IL-6 has been implicated in AD pathogenesis through several different mechanisms. For example, cultured cortical neurons show more damage when treated with both Aβ and IL-6. Further, in cultured hippocampal neurons, IL-6 has been shown to increase tau phosphorylation (Quintanilla et al., 2004).

### AD, Menopause and Hormone Therapy

Since women are disproportionately affected by AD, there has been considerable interest in understanding differences in estrogen across the lifespan associated with AD risk. One approach to investigating this idea is to consider the effects of pregnancies, which yield a net decrease in lifetime estrogen exposure (Bernstein et al., 1985; Hankinson et al., 1995). Several studies have shown that women who have birthed children are more likely to suffer from cognitive impairment and AD than nulliparous women (Ptok et al., 2002; McLay et al., 2003; Colucci et al., 2006; Beeri et al., 2009). Men with or without children show no change in AD risk, so the difference cannot be due to environmental factors associated with child rearing (Ptok et al., 2002). Nulliparous women are therefore seemingly protected from cognitive decline by their greater lifetime exposure to estrogens.

Another approach to address this issue is to consider the potential effects of estrogen loss on AD risk. Most studies have found that surgical menopause *prior* to the development of natural menopause significantly increases risks for cognitive decline and AD (Rocca et al., 2007, 2011; Phung et al., 2010; Bove et al., 2014). Conversely, oophorectomy *after* the age of natural menopause does not appear to significantly alter AD risk (Rocca et al., 2011; Imtiaz et al., 2014). The implication of these studies is that early loss of sex steroid hormones can accelerate the development of AD. However, although brain levels of estrogens are lower in women with AD than age-matched women without neurologic disease, this relationship has been reported for only for women older than age 80 years (Yue et al., 2005; Rosario et al., 2011). Thus, while low estrogen appears to be associated with AD, the timing of this relationship with respect to disease onset remains unsettled.

If the depletion of ovarian sex steroid hormones at menopause is a risk factor for AD, then maintenance of hormones would be predicted to reduce AD risk. Consistent with this idea, AD risk has been reported to be lowest in postmenopausal women with the highest endogenous E2 levels and greatest in those with low E2 levels (Manly et al., 2000). Another approach is to consider how AD risk is affected by treatment with estrogen-based HT. Initial observational studies generally found significantly reduced risk of AD in women with a history of hormone therapy (HT) use (Henderson et al., 1994, 1996), findings that were supported by subsequent prospective studies (Tang et al., 1996; Kawas et al., 1997; Zandi et al., 2002). Human studies have been mirrored by experiments in transgenic mouse models of AD, in which ovariectomy (OVX) induced loss of sex steroid hormones increases and treatment with E2 generally reduces AD-like neuropathology (Zheng et al., 2002; Yue et al., 2005; Carroll et al., 2007; Zhao et al., 2011).

Despite the apparent benefits of estrogen-based HT in reducing AD risk, discrepant clinical findings have demonstrated neural risks and questioned its benefits. Most importantly, results of the Women’s Health Initiative (WHI), a large double-blinded, placebo-controlled clinical trial, indicated increased rather than reduced dementia in subjects randomized to HT treatment (Shumaker et al., 2003, 2004). There are numerous factors that may contribute to the discordance of estrogen’s benefits across the many human and rodent paradigms, including formulation of the treatment, continuous vs. discontinuous delivery, and route of administration. Perhaps the most significant issue is the timing of treatment, with initiation of HT near the onset of menopause hypothesized to be critical for its neural efficacy (Maki, 2006). In the WHI, the mean age of subjects was about 65 years old, many years past the average onset of menopause at age 51. Studies in which HT was initiated at or near menopause have generally reported benefits rather than risks. For example, in a Danish study in which middle aged women were randomized to HT and placebo treatment groups, beneficial effects on cognitive function were observed more than 10 years after the cessation of the 2–3 year HT regimen (Bagger et al., 2005). This finding suggests that using HT, even for a short time during menopause, may have a positive effect on cognition much later. Similar results have been found for AD risk: HT is associated with decreased risk if delivered near menopause but has no benefit or even increases risk when started several years after menopause (Henderson et al., 2005; Whitmer et al., 2011; Shao et al., 2012). More work will be required to fully elucidate the relationship between HT and AD risk, but emerging results seem to suggest that short-term HT near menopause onset may offer a reasonable strategy to impede development of dementia.

Efficacy of HT is also likely to be modulated by genetic factors. The most significant genetic risk factor for late-onset AD is the ɛ4 allele of ApoE4. ApoE4 regulates lipid trafficking and may play a role in the removal of Aβ from the brain (Näslund et al., 1995; Leduc et al., 2011). HT may be differentially effective in women based on their ApoE status. In mice, E2 is able to reduce inflammatory markers in cultures from ApoE3, but not ApoE4 mice (Brown et al., 2008). One study showed that HT around menopause is more effective in preventing cognitive decline in ApoE4-negative women than those with even a single ApoE4 allele (Yaffe et al., 2000). Further, the ApoE4 genotype may increase the risk of AD more in females than males (Farrer et al., 1997; Bretsky et al., 1999). Increased AD risk in ApoE4 carriers requires only one ApoE4 allele in females with no further risk increase in homozygotes, whereas significant male risk appears to require two ApoE4 copies (Payami et al., 1996). A more recent study showed that both male and female ApoE4 carriers were at greater risk of AD with even one ApoE4 allele, but that the risk for female carriers was significantly greater (Altmann et al., 2014). How interactions between ApoE, HT and AD are modulated by obesity, inflammation, and other factors that drive AD pathogenesis remains to be determined.

### Menopause and Adiposity

The shift from young adulthood into middle age is associated with increasing proportions of women that are overweight and/or obese (Ogden et al., 2014). This weight gain likely reflects multifaceted consequences of aging. There is evidence that depletion of sex steroid hormones during menopause can contribute to weight gain, although body weight also predicts subsequent changes in hormone levels (Guthrie et al., 1999; Sternfeld et al., 2004; Wildman et al., 2012). Significant increases in central fat have been reported with greater waist circumference after the last menstrual period (Poehlman et al., 1995; Björkelund et al., 1996; Ho et al., 2010). This increase in adiposity associated with menopause is linked with increased risks for obesity (Rachoń and Teede, 2010), metabolic syndrome (Carr, 2003; Cho et al., 2008) and T2D (Wajchenberg, 2000). Although increasing age may partially account for the increase T2D incidence in postmenopausal women (Janssen et al., 2008), the change in fat distribution has also been shown to be a contributing cause (Barrett-Connor et al., 1996; Tchernof et al., 1998; Sites et al., 2000).

Studies in both humans (menopause) and rodents (reproductive senescence) indicate that age-related ovarian hormone loss contributes to changes in the distribution of adipose tissue. The significant decrease in estrogen and progesterone that results from follicular depletion yields a more androgenic pattern of fat distribution—an increase in central or abdominal adiposity. OVX in mice, a model of surgical menopause, can result in a significant increase in body weight (Stubbins et al., 2012). This elevated weight results from a decrease in energy expenditure, as opposed to increases in calorie consumption, and promotes insulin resistance, increased adipocyte size, and peripheral inflammation (Rogers et al., 2009). Treatment of mice with the E2 results in reductions in all of these outcomes.

In postmenopausal women, estrogen-based HT has been shown to decrease some of these metabolic effects in addition to restoring a more gynoid pattern of fat distribution (Barrett-Connor et al., 1996; Salpeter et al., 2006). HT can attenuate central adiposity when administered to early postmenopausal women (Haarbo et al., 1991; Ahtiainen et al., 2012). Additionally, HT may decrease total weight gain during menopause, especially in non-obese women (Kristensen et al., 1999). Further, HT has been shown to increase the effectiveness of concurrent lifestyle and pharmaceutical treatments for some obesity-related comorbidities (Golden et al., 2013).

The mechanisms by which E2 reduces obesity and risk of T2D remain to be fully elucidated. Paradoxically, risk of maternal insulin resistance is highest in late pregnancy when E2 levels are high (Ryan and Enns, 1988), yet low E2 levels after menopause have been linked with T2D (Carr, 2003; Meyer et al., 2011). Aromatase knockout mice, which cannot synthesize E2, are obese and insulin resistant (Jones et al., 2000). Estrogen receptor α (ERα) is likely to play a role in regulation of obesity as ERα knockout mice are more obese and glucose intolerant than wild-type females (Heine et al., 2000; Bryzgalova et al., 2006). Further, both peripheral and central E2 administrations have been shown to act through different pathways with similar overall outcomes of reductions in insulin resistance, glucose dysregulation and adiposity (Yonezawa et al., 2012). Peripheral and central E2 administration resulted in decreased adipocyte size and reduced expression of macrophage markers, although peripheral E2 treatment elicited greater changes. Only peripheral E2 administration decreased adipose expression of the pro-inflammatory cytokine TNFα. Central and peripheral E2 administration both increased energy expenditure, but central E2 caused much greater spontaneous locomotor activity. Thus, E2 likely regulates adiposity and metabolic outcomes by actions on several tissues. How these actions are affected by aging likely underlies the interactions between menopause, obesity and the efficacy of HT.

### Inflammation and Menopause

There are extensive literatures on the relationships between estrogens and other sex steroid hormones and regulation of inflammation. In general, one can reasonably argue that estrogens function as potent anti-inflammatory factors. Thus, depletion of E2 at menopause results in elevated pro-inflammatory cytokines and may place tissues throughout the body at increased risk of inflammation and diseases associated with inflammation (Pfeilschifter et al., 2002). However, this is an extensive and complex literature as estrogens regulate several aspects of immune function and inflammation (Straub, 2007) and thus can protect against some conditions but promote others (Gilliver, 2010). The relationships between estrogens, menopause, and inflammation-related disorders have been well described in numerous reviews for several conditions, including cardiovascular disease (Camilleri et al., 2012; Knowlton and Lee, 2012), osteoarthritis (Martín-Millán and Castañeda, 2013), and rheumatoid arthritis (Islander et al., 2011). Thus, although estrogens can be broadly defined as anti-inflammatory, the effects of estrogen loss and estrogen-based HT depend upon the tissue, cell type, and context.

How menopause affects neuroinflammation has not been well studied. However, as with other tissues, the brain exhibits a generally pro-inflammatory phenotype with increasing age. In a study that examined changes in gene expression across age in adult human brain, male and female brains exhibited significant differences in the number of genes changing expression (higher in males), the patterns of changes in terms of gene function, and the brain region-specific nature of the changes (Berchtold et al., 2008). Of particular interest to the current topic, women exhibited greater proportional age-related increases in expression of genes associated with immune and inflammatory functions. Further, both women and men showed increased expression of these genes in hippocampus and entorhinal cortex (both of which are strongly affected in AD), but only women had significant increases in other brain regions (Berchtold et al., 2008), suggesting a more global pro-inflammatory condition in the aging female brain.

Like the human brain, the rodent female brain shows increased inflammation with aging that is regulated, in part, by estrogen status. For example, the frontal cortex of middle-aged female rats shows increased expression of several microglial and immune function genes as a consequence of both aging and estrogen-deprivation (Sárvári et al., 2012). Importantly, the authors also found a strongly overlapping pattern of gene expression changes in the frontal cortex of older postmenopausal women relative to younger premenopausal women. Confirming a protective role of estrogens, treatment of OVX middle-aged rats with E2 or ER-specific agonists significantly decreased expression of several microglial and immune function genes in the frontal cortex (Sárvári et al., 2011). This relationship extends to pro-inflammatory cytokines such as TNFα and IL-1β, which show increased hippocampal expression in female mice as consequences of both aging and OVX-induced estrogen depletion (Benedusi et al., 2012). The age-related increase in the pro-inflammatory state of the brain can be exacerbated by inflammatory challenges. For example, the increased hippocampal expression of cytokines induced by high cholesterol diet is significantly worsened in reproductively senescent female rats, an effect that can be attenuated by estradiol treatment (Lewis et al., 2010). However, the ability of E2 to protect against elevated cytokine levels can significantly diminish, and in some cases reverse, in aged female brain (Nordell et al., 2003). Collectively, these observations indicate that both aging and menopause contribute to increasing levels of neuroinflammation, which are predicted to cooperatively interact in the promotion of inflammation neural diseases such as AD.

## Conclusion

**Figure 1:**
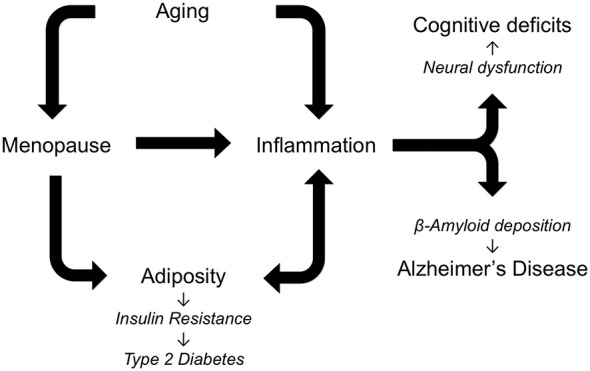
**Alzheimer’s disease (AD) is a multifactorial disorder in which multiple risk factors are theorized to interact in regulating pathogenesis.** As depicted in the diagram an essential factor in AD is increasing age, which is also associated with elevated inflammation and, in women, menopause. The loss of estrogens at menopause increases central adiposity, which in turn increases inflammation and predisposes women to metabolic syndrome, insulin resistance, and AD. Individually and cooperatively, aging, menopause, adiposity, and inflammation lead to cognitive deficits and AD.

AD neuropathology is characterized and likely driven by the accumulation of Aβ and abnormally phosphorylated tau. The development of these and other components of AD neuropathology result from the interactive effects of several risk factors (Figure 1). Most importantly, AD is dependent upon aging. The negative outcomes of all genetic and environmental risk factors of AD require advancing age. In women, chronological aging is also tied to reproductive aging that is manifested as menopause in mid-life. Menopause results in cessation of the ovarian cycle and its cyclical production of estrogens and progesterone, sex steroid hormones with numerous protective roles against AD (Pike et al., 2009). Beyond protective actions against specific aspects of AD neuropathology, estrogens function throughout the body to guard against the development of increasing central adiposity and inflammation, both of which are implicated in the initiation and progression of AD. Of course, aging is also associated with significant increases in inflammation and central obesity, independent of estrogen levels. Thus, women are simultaneously exposed to the consequences of both chronological and reproductive aging, both of which can function as drivers of AD, obesity, and inflammation.

Estrogen-based HT initiated prior to the end of menopause has been shown to be effective in combating both the increased adiposity and the cognitive decline associated with aging. Although HT can exhibit adverse side effects, especially in those women who may be predisposed, emerging results suggest that relatively short-term HT initiated near the onset of menopause is likely to yield largely beneficial effects for most women. Much more research will need to be done to determine precisely which genetic or environmental factors define the target population.

Inflammation is another seemingly invariant consequence of aging. In females, inflammation is further increased by the loss of estrogens at menopause. As with many other age-related diseases, AD is linked with elevated inflammation during aging by abundant data from several fields. Several specific inflammatory factors have been linked to both obesity and AD including leptin, TNF-α and IL-6. Since estrogens protect against both the development and deleterious consequences of inflammation across many tissues, depletion of estrogens at menopause is theorized to contribute to women’s vulnerability to AD. Age-related increases in adiposity and changes in the distribution of fat to central depots further increase the risk of obesity, which promotes downstream conditions including insulin resistance, metabolic syndrome and T2D. Thus, a highly interactive set of relationships develops in women: the cumulative effects of aging, menopause, adiposity, and inflammation result in increased risk for neural dysfunction and AD pathogenesis (Figure 1). Certainly this set of factors is incomplete, as new data continue to identify additional factors, including bioenergetics, that not only are altered during reproductive aging but also can promote AD (Yin et al., 2015). Continued research is needed to address many unresolved issues about these pathological interactions, including how they are modified by genetic risk factors such as Apolipoprotein E (ApoE) and the extent to which they can be effectively attenuated by estrogen-based HT.

## Conflict of Interest Statement

The authors declare that the research was conducted in the absence of any commercial or financial relationships that could be construed as a potential conflict of interest.
